# Prevalence and species identification of *Cryptosporidium* spp. in the newborn dairy calves from Muang District, Khon Kaen Province, Thailand

**DOI:** 10.14202/vetworld.2019.1454-1459

**Published:** 2019-09

**Authors:** Phennarin Doungmala, Patchara Phuektes, Weerapol Taweenan, Somboon Sangmaneedet, Ornampai Japa

**Affiliations:** 1 Interdisciplinary Veterinary Science, Faculty of Veterinary Medicine, Khon Kaen University, Khon Kaen 40002, Thailand; 2 Division of Pathobiology, Faculty of Veterinary Medicine, Khon Kaen University, Khon Kaen 40002, Thailand; 3 Department of Microbiology and Parasitology, Faculty of Medical Sciences, Phayao University, Phayao 56000, Thailand

**Keywords:** *Cryptosporidium*, Khon Kaen Province, nested polymerase chain reaction, newborn dairy calves, prevalence

## Abstract

**Aim:**

This study aims to determine the prevalence of *Cryptosporidium* spp. infection and to identify the species of *Cryptosporidium* spp. in newborn dairy calves between December 2016 and March 2017 in Muang District, Khon Kaen Province, Thailand.

**Materials and Methods:**

A total of 200 fecal samples from newborn dairy calves of the ages 1 day up to 28 days were collected and the presence of *Cryptosporidium* oocysts was examined microscopically using the modified Kinyoun’s acid-fast staining technique. Then, *Cryptosporidium* species were identified using nested polymerase chain reaction amplification of 18S rRNA gene and sequencing.

**Results:**

The modified Kinyoun’s acid-fast staining revealed the presence of *Cryptosporidium* oocysts in 51% (102/200). Sequence analysis of the 18S rRNA gene identified two species, namely, *Cryptosporidium* *bovis* (n=11) and *Cryptosporidium* *ryanae* (n=11) and one isolated strain could not be identified.

**Conclusion:**

This study indicated that newborn dairy calves aging up to 4 weeks were highly infected with *Cryptosporidium* spp., and the infection mostly occurred in diarrheic dairy calves. This is the first report of *Cryptosporidium* in dairy calves in Khon Kaen Province and the results provide baseline information for further studies and control of *Cryptosporidium* infection in dairy calves in the study area.

## Introduction

Cryptosporidiosis is an important zoonotic disease caused by *Cryptosporidium* spp. transmitted among humans and animals. The disease is distributed worldwide including Thailand and has been found in cattle, goats, sheep, horses, pigs, dogs, cats, mice, chickens, turkeys, ducks, fishes, reptiles, wildlife, and in humans [1-3]. *Cryptosporidium* causes diarrhea in young animals due to their low immunity [4], especially in newborn dairy calves of 1-3 weeks of age [5]. The most important *Cryptosporidium* species causing economic losses is *Cryptosporidium parvum*. *Cryptosporidium* spp. parasitize the intestinal epithelium of mammals and other vertebrate hosts causing diarrhea similar to coccidian infection. The infected hosts shed *Cryptosporidium* oocysts in their feces [6]. Disease transmission mainly occurs through ingestion of water and food contaminated with oocysts [7]. Clinical signs of infected hosts depend on their immune status. Diarrhea may be in hosts having a strong immunity to the protozoa recover from diarrhea spontaneously. Clinical signs of cryptosporidiosis in neonatal dairy calves may vary from none to severe diarrhea accompanied by other signs such as depression, anorexia, fever, and severe dehydration [7]. *Cryptosporidium* infections in humans and animals are often neglected, with attention being paid only when hosts suffer from chronic severe diarrhea. Oocysts of *Cryptosporidium* can be detected in the feces of the immunosuppressed calves and humans. People working in dairy farms may be infected with *Cryptosporidium* through ingestion of food and water contaminated with oocysts. The infected hosts excrete oocysts in diarrheal feces [6].

Four major species of *Cryptosporidium* have been described as the bovine pathogen; *Cryptosporidium andersoni, Cryptosporidium bovis*, *C. parvum*, and *Cryptosporidium ryanae* [8-10]. Although it is rare, some other *Cryptosporidium* species have been found in cattle; *Cryptosporidium felis* in cow [11], *Cryptosporidium hominis* in a 3-day-old calf and 6-year-old cow from Scotland [12], and *Chlamydia suis* in a calf [11,13]. In livestock, only *C*. *parvum* is associated with clinical signs of unweaned calves (less than 6 weeks), *C. bovis* and *C. ryanae* are generally more common than *C. parvum* in post-weaned calves (1.5 months-11 months) without causing disease or any clinical signs. *C. andersoni* is considered the main pathogenic species, causing sporadic cases and outbreaks in the digestive glands of abomasum of older calves and adult cattle and has been shown to reduce dairy production. Infected cattle do not develop diarrhea but show clinical signs of reduced weight gain and milk yield and can excrete oocysts for several months.

The occurrence and species infection rate of *Cryptosporidium* infections in Thailand are not thoroughly investigated. Due to its zoonotic potential, this study aims to reveal the incidence of *Cryptosporidium* infection in dairy calves from Muang District, Khon Kaen Province and describes its association with age groups and identifies species of *Cryptosporidium* spp. The results of this study may provide useful information for the future research on prevention, control, and treatment of this important neglected disease in newborn dairy calves.

## Materials and Methods

### Ethical approval

The present study was approved by the Animal Ethics Committee of Khon Kaen University, based on the Ethics of Animal Experimentation National Research Council of Thailand.

### Fecal sample collection and examination

A total of 200 fecal samples from 49 Holstein dairy farms were collected, classified according to their age Group I (1-7 days) 50 samples, Group II (8-14 days) 50 samples, Group III (15-21 days) 50 samples, and Group IV (22-28 days) 50 samples and were taken randomly from eight villages (Noen Thong, Sumjan, NonLan, Khok Si, Hin Lad, Non, Subcharoen, and Kham Bon) of two subdistricts of Muang District, Khon Kaen Province, between December 2016 and March 2017. Subsequently, the fecal samples were collected directly from rectum using disposable latex gloves and kept in the labeled cleaned polyethylene bags and brought to the laboratory and refrigerated at 4°C until examined (for a maximum of 3 days).

Fecal samples were examined microscopically using the modified Kinyoun’s acid-fast staining technique (cold method, with reagent: Carbol fuchsin and methylene blue) for the detection of *Cryptosporidium* oocysts. With this cold Kinyoun’s acid-fast method, *Cryptosporidium* oocysts will be stained pink to red to deep purple on a pale blue background, measuring 4-6 µm in diameter and some of the four sporozoites may be visible [14]. All fecal samples were stored at −20°C, without preservatives until used.

Total DNA was extracted from fecal samples using QIAamp DNA Stool Mini Kit (Qiagen, GmbH, Germany), and species identification of *Cryptosporidium* was performed using nested polymerase chain reaction (nested PCR) analysis of 830 bp fragment of the 18S rRNA gene [15]. The secondary PCR products were visualized using 1.5% agarose gel electrophoresis. PCR products were purified using QIAquick PCR Purification Kit (Qiagen, GmbH, Germany). Sequencing was performed in an automated DNA sequencer, the BigDye^™^ Terminator v3.1 Cycle (Axil Scientific Pte Ltd., The Gemini, Singapore Science Park II, Singapore). The results of DNA sequences were compared with those in GenBank database, using the BLAST program (www.ncbi.nlm.nih.gov/blast).

Results were analyzed using Epi Info version 7.0 (CDC, USA). Descriptive statistics were used to determine the infection rate of *Cryptosporidium* infection in dairy calves. Comparison of infection rate of *Cryptosporidium* infection in different groups of dairy calves (location, age) was conducted using Chi-square test. p<0.05 was considered statistically significant.

## Results

### *Cryptosporidium* infection rate in different age groups of newborn dairy calves

Of 200 fecal samples from dairy calves, 102 (51%) were *Cryptosporidium* positive by modified Kinyoun’s acid-fast stain. Geographical variation of the infection rate is shown in Table-1.

**Table 1:** *Cryptosporidium* spp. infection rate of newborn dairy calves in eight villages.

Location	Microscopic detection (%)	Nested PCR detection (%)
Non Thon subdistrict		
Hin Lad	0/2 (0)	1/2 (50.0)
Non	2/8 (25.0)	1/8 (12.5)
Subcharoen	5/8 (62.5)	2/8 (25.0)
Kham Bon	8/11 (72.7)	3/11 (27.3)
Subtotal	15/29 (51.7)	7/29 (24.1)
Ban Khao subdistrict		
Noen Thong	35/53 (66.0)	3/5 (5.7)
Sumjan	11/46 (23.9)	8/46 (17.4)
Non Lan	35/63 (55.6)	13/63 (20.6)
Khok Si	6/9 (66.7)	3/9 (33.3)
Subtotal	87/171 (50.9)	27/171 (15.8)
Total	102/200 (51.0)	42/200 (21.0)

PCR=Polymerase chain reaction

Chi-square test, there was a significant difference in proportions of positive dairy calves among different villages and subdistricts (p<0.05).

*Cryptosporidium* spp. infection in 1-7-day-old dairy calves in six different regions of Muang District, Khon Kaen Province, was found almost all regions except non-village. The overall infection rate of *Cryptosporidium* spp. was 50% (0-80%) in this age group, and the infection rate of *Cryptosporidium* spp. was found highest in Khok Si village (80%). In 8-14-day-old dairy calves in seven different regions of Muang District, Khon Kaen Province, the infection of *Cryptosporidium* spp. was found almost in all regions except Non-village and Kham Bon village, and the overall infection rate of *Cryptosporidium* spp. was 44% (0-100%). In 15-21-day-old dairy calves in six different regions of Muang District, Khon Kaen Province, the infection of *Cryptosporidium* spp. was found almost in all regions except Non-village and Subcharoen village, and the overall infection rate of *Cryptosporidium* spp. was 60% (0-100%). In 22-28-day-old group in seven different regions of Muang District, Khon Kaen Province, the infection of *Cryptosporidium* spp. was found highest almost all regions except Sumjan village, and the overall infection rate of *Cryptosporidium* spp. was 48% (0-100%) (Table-2). The infection rate of *Cryptosporidium* spp. of newborn calves of different age ranged from 44% to 60% by microscopic examination, whereas that by nested PCR detection ranged 16-26% (Table-2). Chi-square test conferred no significant difference in the infection rate of dairy calves of different age groups (p>0.05).

**Table 2:** Prevalence of *Cryptosporidium* spp. in each age group of newborn dairy calves.

Age group	Microscopic detection (%)	Nested PCR detection (%)
Group I (1-7 days old)	25/50 (50.0)	13/50 (26.0)
Group II (8-14 days old)	22/50 (44.0)	12/50 (24.0)
Group III (15-21 days old)	30/50 (60.0)	9/50 (18.0)
Group IV (22-28 days old)	24/50 (48.0)	8/50 (16.0)

PCR=Polymerase chain reaction

### Identification of *Cryptosporidium* species by 18s rRNA sequencing

The molecular analysis of 200 fecal samples revealed that 42 (21%) were positive for *Cryptosporidium*, which was far less than positive rate by microscopic analysis 51%. By nested PCR, 42 samples were successfully amplified and were sequenced later. Sequence analysis from 23 readable electrophoregrams revealed that *Cryptosporidium* in the present study had 83-99% similarities with genotypes listed in Genbank: *C. ryanae* (11 strains) was detected in all age groups and found in almost every village except Khok Si, Non, and Subcharoen; *C. bovis* (11 strains) was detected only in age Groups I, II, and III and found in almost every village except Khok Si, Hin Lad, Non, and Kham Bon; and one unidentified species of *Cryptosporidium* spp. was detected in age Group I in Noen Thong. The rest of other *Cryptosporidium* nested PCR products demonstrated weak bands that could not be sequenced. *C. parvum* and *C. andersoni* were not found in this study (Table-3).

**Table 3:** Results of genotypic identification of *Cryptosporidium* spp. and accession against GenBank database.

Species of *Cryptosporidium*	Accession and identity with GenBank (%)	Location	Age group
*Cryptosporidium* spp. (full sequence)	KF994564 (99)	Noen Thong	Group I
*C. ryanae* (full sequence)	KJ020908 (99)	Hin Lad	Group I
*C. ryanae* (full sequence)	KP793013 (99)	Sumjan	Group I
*C. bovis* (partial sequence)	MG972763 (99)	Non Lan	Group I
*C. bovis* (full sequence)	KF128742 (99)	Non Lan	Group I
*C. bovis* (full sequence)	HQ179573 (99)	Non Lan	Group I
*C. ryanae* (partial sequence)	KJ020908 (98)	Sumjan	Group I
*C. bovis* (partial sequence)	MG972763 (98)	Subcharoen	Group II
*C. bovis* (partial sequence)	MG972763 (92)	Subcharoen	Group II
*C.ryanae* (partial sequence)	MF671875 (99)	Sumjan	Group II
*C. bovis* (partial sequence)	MG972763 (99)	Sumjan	Group II
*C. ryanae* (partial sequence)	KJ020908 (99)	Non Lan	Group II
*C. ryanae* (partial sequence)	KJ020908 (99)	Kham Bon	Group II
*C. ryanae* (partial sequence)	MF671875 (97)	Non Lan	Group II
*C. ryanae* (partial sequence)	KJ020908 (99)	Non Lan	Group II
*C. bovis* (partial sequence)	MG972763 (97)	Noen Thong	Group II
*C. bovis* (partial sequence)	MG972763 (97)	Noen Thong	Group II
*C. ryanae* (partial sequence)	KJ020908 (99)	Sumjan	Group II
*C. bovis* (partial sequence)	MG972763 (97)	Non Lan	Group II
*C. ryanae* (partial sequence)	MF671876 (98)	Noen Thong	Group III
*C. bovis* (partial sequence)	MG972763 (99)	Noen Thong	Group III
*C. bovis* (partial sequence)	MG972763 (83)	Non Lan	Group III
*C. ryanae* (full sequence)	MF671875 (97)	Sumjan	Group IV

*C. ryanae=Cryptosporidium ryanae*, *C. bovis*=*Cryptosporidium bovis*

## Discussion

In the study area, newborn dairy calves of age up to 4 weeks were highly infected with *Cryptosporidium* spp. The infection mostly occurred in diarrheic dairy calves, suggesting that, *Cryptosporidium* may be a major causative agent of diarrhea in young calves aged up to 4 weeks. *Cryptosporidium* infection causes retarded growth of calves or induces secondary infections with other microorganisms. Young calves or immunocompromised animals are susceptible to infection with *Cryptosporidium* spp. than adults [2,4,5]. A relatively high infection rate of *Cryptosporidium* spp. infection has been detected in dairy calves elsewhere, with the oocysts being detected, especially in dairy calves aged 1-3 weeks [5]. Highest shedding of *Cryptosporidium* oocysts occurred in dairy calves aged 3 weeks. The present study was the first identification of *Cryptosporidium* spp. infection in dairy calves in Muang District, Khon Kaen Province. In a total of 200 dairy calves investigated, microscopically positive with *Cryptosporidium* spp. (102/200 [51%]) was higher than nested PCR (42/200 [21%]), this may cause by inhibitors in feces that inhibit the activity of the enzyme; therefore, DNA could not be increased during the PCR process. The microscopy results were higher than previous studies in Thailand, i.e., 12.63% in Chiang Mai and Saraburi [16], 0.6% in Nong Pho region of Central Thailand [17], 5% in Chiang Mai, Chiang Rai, and Lumpang [18], and 0.5% in Saraburi, Kanchanaburi, and Nakornpathom [19]. The infection rate of *Cryptosporidium* detected by nested PCR was higher than previous studies in Thailand, i.e., 7.6% and 1.3% [18,19]. The results of this study were in line with those previously reported in the other countries, the rates of *Cryptosporidium* spp. infection in pre-weaned dairy calves, 41.5% with being reported in France [20], 10.2% in England [21], 25.2% and 17.3% in Ethiopia [22,23], 6.9% in Egypt [24], 27.10% in Eastern Algeria [25], 78.1% in Nigeria [26], 22.3%, 3.6%, and 24-31% in Iran [27-29], 7.3% in Iraq [30], 1.39%, 15.6%, 14.4%, and 6.4% in China [31-34], 43.3% in Vietnam [35], 82% in Myanmar [36], 3.2% in Malaysia [37], and 37.25% in India [38]. Age of animal is an important factor in infection with *Cryptosporidium* spp., for example, calves are commonly more susceptible than cows to infection [39]. The subclinically infected ruminants and cows can be carriers and transmit *Cryptosporidium* spp. to calves. The variance of infection rates depended on the geographical and environmental variations, management of cares, and hygiene in the farms [40]. The high rate of *Cryptosporidium* infection in newborn dairy calves in this study indicates that more attention should be paid to *Cryptosporidium* spp. in dairy farms to prevent diarrhea in young animals and their potential transmission to humans.

Species of *Cryptosporidium* were determined in calves and some of them have been identified as zoonotic pathogens [41]. The results of the genotyping of *Cryptosporidium* spp. using nested PCR and DNA sequencing revealed that some samples in this study were positive for *C. bovis* and *C. ryanae*, both of which can infect only cattle, although they are not pathogenic to cattle. In this study, *C. andersoni* was not detected. This species has been reported in adult cattle only and rarely detected in the pre-weaned animals [21,42]. *C. parvum*, which is known as zoonotic species and infects both farm animals and humans [43], was also not found in this study. In Thailand, the presence of *C. parvum* and *C. bovis* in cattle was demonstrated by sequencing analysis in the previous study [18,19]. Since asymptomatic cattle infected with *C. parvum* can be the main reservoir of disease, we should pay attention to the control and prevention of this infection both in humans and animals to avoid mutual transmission between humans and animals.

This is the first study of infection rate of *Cryptosporidium* spp. in dairy calves in Muang District, Khon Kaen province, Thailand. This study revealed the overall *Cryptosporidium* infection rate of 51% (102/200) with microscopic examination and 21% (42/200) with nested PCR. Genotype analysis revealed that only two species, *C. ryanae* and *C.bovis*, were present in the study area. Since the variety of species of *Cryptosporidium* in Thailand is still unclear in each region, baseline information for the diversity of this organism with molecular epidemiology in relation to clinical symptoms is required for the control of *Cryptosporidium* infection.

## Conclusion

This study is the first report of *Cryptosporidium* infection in the newborn dairy calves in Khon Kaen province, Thailand. The newborn dairy calves aging up to 4 weeks were highly infected with *C. ryanae* or *C. bovis*, and diarrhea was associated with the *Cryptosporidium*-infected dairy calves.

## Authors’ Contributions

PD collected the samples, analyzed the data, and wrote the manuscript. PP and OJ applied PCR testing. WT and SS designed the study and approved the final manuscript.
